# Degradation of pigments in *Limnospira platensis* extracts

**DOI:** 10.1002/jsfa.70540

**Published:** 2026-03-16

**Authors:** Marisa Cardoso, Carla Brazinha, Carla AM Portugal

**Affiliations:** ^1^ LAQV REQUIMTE, Chemistry Department, NOVA School of Science and Technology | NOVA FCT Universidade Nova de Lisboa Caparica Portugal

**Keywords:** chlorophyll, enzymatic oxidation, food ingredient, microalgae, nutraceutical, peroxidase, UV‐Vis spectroscopy

## Abstract

**BACKGROUND:**

*Limnospira platensis*, commonly known as spirulina, holds promise for application as a food ingredient and nutraceutical due to its rich protein and antioxidant content, including chlorophylls, carotenoids and phycocyanin. Despite its potential, the vibrant colour poses a challenge for consumer acceptance, hampering the marketability of spirulina‐based products. To overcome this, innovative strategies are needed to effectively reduce colour while preserving its nutritional value.

**RESULTS:**

This study sought to compare the impact of endogenous enzymatic and exogenous horseradish peroxidase (HRP)‐mediated processes on colour degradation in spirulina extracts. Intrinsic and extrinsic colour degradation experiments were conducted, with and without hydrogen peroxide (H_2_O_2_ ) and 4‐hydroxybenzenesulfonic acid (PSA) as co‐substrate and analysed using UV–visible spectroscopy. Specifically, the absorbance changes were monitored at 440 and 677 nm, corresponding to chlorophylls *a* and *b*, the predominant pigments in spirulina extracts. The degradation of spirulina extracts exhibited a distinct two‐stage pattern. The initial stage, observed within the first hour, displayed a rapid decrease in absorbance, followed by a slower decline in the subsequent stage. In the presence of H_2_O_2_ , intrinsic degradation caused a gradual absorbance reduction of less than 30% after 24 h. Importantly, the introduction of exogenous factors, including HRP and PSA, resulted in a remarkable 40% absorbance decrease within less than 30 min.

**CONCLUSION:**

This substantial enhancement in decoloration efficiency, surpassing the intrinsic metabolic activity, underscores the potential of extrinsic enzymatic approaches. These findings hold promise for addressing the challenge of spirulina extract colouration in products, potentially enabling improved market acceptance and viability. © 2026 The Author(s). *Journal of the Science of Food and Agriculture* published by John Wiley & Sons Ltd on behalf of Society of Chemical Industry.

## INTRODUCTION

*Limnospira platensis*, commonly known as spirulina platensis or just spirulina, is a species of a blue‐green cyanobacteria.^1^ Cyanobacteria are a phylum of microorganisms related to bacteria but capable of converting solar energy into chemical energy through photosynthesis. For this reason, they are generally included into the group of microalgae.^2^, ^3^ Spirulina can be easily cultivated in water, growing in sunlight, at hight temperatures and has high tolerance to alkaline pH.^4^ These characteristics make it easy to populate in various environments such as soils, lakes, brackish, marine and sweet water.^5^ Spirulina has a morphological spiral shape of cylindrical filaments forming an open helix. The surface cells are smooth and the soft cell walls are easily broken, so its easily digestible by simple enzymatic systems.^4^, ^6^

Spirulina has been revealed to be a great source of valuable food ingredients and nutraceutical compounds finding promising applications in the food and pharma industries. For this reason, spirulina has received increased attention from researchers due to its economical, ecological and nutritional importance.^4^, ^7^ Spirulina has been massively cultured, representing over 30% of the world's microalgal biomass production^2^ and it is considered as a ‘superfood’. The recognition of spirulina as an important food supplement is mainly due to its high protein content (around 60–70% w/w) and its richness in vitamins (4% w/w), minerals, antioxidants, essential amino acids and essential fatty acids.^8^, ^9^, ^10^ Is also an important source of other value‐added compounds such as chlorophyll, carotenoids and phycobiliproteins like phycocyanin.^2^ These abundant and diverse functional compounds that can be extracted from spirulina are known to exert potential health benefits. For example, carotenoids exhibit provitamin A activity and their consumption has been associated with the reduction of risk of developing degenerative chronic diseases and some types of cancer and with the enhanced immune system.^11^ Several other studies have reported the use of spirulina species to regulate diabetic processes and have antioxidative effects and radical scavenging properties, which can provide important multiorgan protection.^1^ Phycocyanin is used in the cosmetics, food and pharmaceutical industries because of its antioxidant, anti‐inflammatory and anticancer properties.^12^ Spirulina has been used as a nutrient for human and animal consumption as tablets, capsules, powder and added either as a nutritional supplement or as a natural colorant.^1^

In the food industry, colour is an important criterion for consumption, as it affects food appearance and consequently consumers’ perception of taste and overall product acceptance. Despite microalgal colouring molecules (pigments) being natural, they may impair the visual attractiveness of products to more traditional consumers. Several decolouration methods have been reported, not only in the microalgal field but also in fruits and vegetables.^13^, ^14^, ^15^

Natural decolourisation methods relying on the intrinsic oxidative metabolism of biomass are generally inefficient, showing low decolourisation/pigment degradation rates. This intrinsic degradation may involve enzymes which are naturally part of the microalgae composition, such as chlorophyllase and endogenous peroxidases, as well as nonenzymatic oxidation processes prompted by reactive oxygen species present in the extracts. The efficiency of these processes is often limited by low enzyme concentrations, suboptimal conditions and the presence of antioxidant substances, such as the pigments which inhibit/retard decolouration processes. These natural decolouration systems have evolved for controlled metabolic processes, and not for fast decolourisation as required for an efficient industrial‐scale application. Therefore, to achieve efficient decolourisation, the addition of an extrinsic enzymatic system is necessary. Such a system can operate independently of the biomass native metabolism, using optimised conditions and powerful, nonspecific oxidative radicals to rapidly target pigments.

In 1974, Lewell and Brown tested solvent extraction, chemical bleaching, intense light treatment and enzymatic processes to decolourise spirulina. Reported was the ability of solvent extraction for colour removal. It showed an inability for a complete colour removal and affected the nutritional value of the product. Light treatment using an intense light source allowed for the decolouration of spirulina extracts, but it led to intense oxidation of cell components and to a substantial decrease in the final protein content.

Critically, these results highlight the need to replace nonspecific methods, which often lead to the degradation of other valuable light‐ and oxygen‐sensitive bioactive compounds, such as phycocyanin and carotenoids, thereby diminishing the overall nutritional and functional value of the extract, by other more selective decolouration processes, such as enzymatic processes. Enzymes may efficiently lead to colourless products simultaneously preserving the functional and nutritional properties of these compounds. Thus, the degradation of these compounds by the decolouration process should not be overlooked but considered a descriptor of process selectivity and efficiency.

Although Lewell and Brown reported the successful decolouration by enzymatic treatment with chlorophyllase, a 30‐day reaction time was necessary to ensure a complete colour removal.^13^

Studies using enzymatic systems for degradation of colour from chlorophyll pigments have also been done in leaves and horticultural crops. Yamauchi and Watada studied the degradation of chlorophyll using the peroxidase–hydrogen peroxide system of leafy vegetables in the presence of 16 commercial phenolic compounds. Chlorophyll was extracted by solvent methods, followed by a reaction of 5 min at 25 °C and then spotted by the addition of a mixture of ethanol and hexane. Samples of this reaction were analysed by HPLC and it was concluded that chlorophyll degradation occurred with apigenin, apigetrin, naringenin, resorcinol and *p*‐coumaric acid.^14^ Years later, Yamauchi reported similar results in horticultural crops and found that not all phenolic compounds effectively degrade chlorophyll in the peroxidase–hydrogen peroxide system, apparently because of their molecular configuration.^16^ The mechanism involved in peroxidase‐mediated chlorophyll degradation into colourless compounds is the oxidation of the phenolic compound with hydrogen peroxide forming a phenoxy radical.^14^, ^16^

The present work aimed at understanding the added effect of extrinsic enzymatic degradation of spirulina pigments, by horseradish peroxidase (HRP), to that obtained by using the intrinsic metabolic system of spirulina. Extrinsic pigment degradation is carried out by enzymes which are not naturally present in microalgae and thus added to the microalgal extracts. Intrinsic pigment degradation would result from the action of enzymes naturally present in microalgae containing chlorophyll species, such as chlorophyll‐chlorophyllido Chlase, chlorophyllase, chlorophyll degrading peroxidase, pheide a oxygenase and red chlorophyll catabolite reductase.^17^, ^18^ These enzymes in the presence of oxidising species, i.e. oxygen species formed in microalgal extracts or present in the air, may contribute to chlorophyll degradation. Ultimately, the study aimed to clarify the advantages of using an external enzymatic process to ensure an efficient decolouration of microalgal extracts.

## EXPERIMENTAL

### Materials

A4F, Algae for Future (Portugal), kindly provided protein extracts from ruptured spirulina biomass. The biomass was cultivated by our industrial partner under proprietary conditions; therefore, specific details regarding strain source and cultivation parameters are not disclosed. The extracts were produced in a small‐scale batch culture and kept at 4 °C in the dark until use. The characteristics of the extract in terms of protein content, pH and conductivity are indicated in Table 1. HRP (lyophilised powder, ~150 units mg^−1^ protein) was supplied by Sigma‐Aldrich and used without further purification. 4‐Hydroxybenzenesulfonic acid (PSA; 65 wt%), sodium phosphate dibasic, bovine serum albumin, Bradford reagent–brilliant blue G and sodium tartrate were purchased from Sigma‐Aldrich. Sodium chloride, potassium chloride and sulfuric acid were purchased from Honeywell Fluka. Potassium phosphate monobasic was purchased from Chem‐Lab. Hydrogen peroxide (3%) was purchased from Dimor Lusitana Lda and sodium hydroxide was supplied by Fisher Scientific.

**Table 1:** Spirulina extract characteristics, namely pH, conductivity and protein concentration^a^

	pH	Conductivity (mS cm^−1^)	Protein concentration (g DW L^−1^)
Spirulina extract A	8.89	7.89	0.52
Spirulina extract B	9.03	17.12	0.18
Spirulina extract C	9.09	22.58	0.69

^a^These data were kindly provided by Algae for Future, A4F.

### Methods

#### Intrinsic degradation of spirulina extracts

The intrinsic degradation of spirulina extracts was evaluated based on spectral changes, observed over 24 h (1140 min) for fresh spirulina extracts or extracts stored at 4 °C, for a short period (typically <48 h) to minimise pre‐assay degradation due to exposure to the atmosphere conditions, i.e. air exposure), at pH 9 and upon adjustment of the media at pH 6.5 and 7. The adjustment of pH was done by adding small volumes of HCl (0.1 mol L^−1^) to the spirulina extract. It was not possible to decrease the extract pH below 6.5. The observed resistance of the spirulina extracts to pH change was possibly due to a strong buffer effect by the microalgal media. The cultivation requirements of spirulina are well established and extensively described in further literature,^19^ confirming that spirulina growth media are characterised by a strong buffering effect at alkaline pH values. Media alkalinity exerts a protective effect avoiding the contamination of spirulina growth media by other microorganisms while contributing to the fixation of specific essential microalgal nutrients, such as ammonia. The effect of sonication on the degradation of spirulina extracts was also studied. In this case, spectral changes were investigated for spirulina extracts after sonication under different conditions. Spirulina extracts were subjected to 3 or 5 sonication cycles, with duration time varying from 20 to 30 s and exposed to sonication amplitudes of 20% and 40%, as described in Table 2. Sonication was applied in pulses to spirulina extract samples immersed in ice baths necessary to avoid extract degradation due to sample overheating.

**Table 2:** Conditions used in sonication of spirulina extract, namely number of cycles, cycle time, time off cycles and sonication amplitude

	Cycles	On	Off	Amplitude
Sonication A	3×	20 seg	5 seg	20%
Sonication B	3×	20 seg	5 seg	40%
Sonication C	5×	30 seg	30 seg	20%

The effect of H_2_O_2_ on the spectral changes was also evaluated to access its effect on the degradation of spirulina extracts. Different amounts of H_2_O_2_ were added to spirulina extracts to vary H_2_O_2_ concentrations up to 76 mmol L^−1^. The extracts were then incubated at room temperature (25 °C) over the experiment time. Aliquots of 200 μL were taken from the extracts over a period of 1440 min for absorbance readings using a microplate reader (Multiskan GO from ThermoScientific).

The absorbance changes at wavelengths of 440 and 677 nm, corresponding to the maximum absorbance of chlorophylls *a* and *b*, were plotted as a function of the reaction time. The pigment degradation rates were elicited based on the absorbance decreasing rates, calculated by determining the slope in the first (*t* < 60 min) and second reaction stages (*t* > 60 min), corresponding to the pigment degradation rates in each of these stages. All the reported experiments were performed with a minimum of three independent replicates.

#### Degradation of spirulina extracts by horseradish peroxidase

The degradation of spirulina extracts was also attempted by the addition of a peroxidase – horseradish peroxidase (HRP) – extrinsic to the spirulina extract composition. The degradation assays by HRP were conducted in the presence of different concentrations of H_2_O_2_, which acted as the electron acceptor, and different concentrations of PSA, which was used as a co‐substrate of the enzymatic reaction. The concentrations of H_2_O_2_ and PSA were varied from 8 to 76 mmol L^−1^, and from 3 to 5.8 mmol L^−1^, respectively. The absorbance measurements were made using the microplate reader mentioned above.

The absorbance spectra were monitored at frequent intervals over a period of 1440 min. Measurements were performed immediately before the addition of HRP to a final concentration of 7.1 μmol L^−1^, H_2_O_2_ and PSA. All measurements were made in triplicate. The degradation rates were assessed by determination of the slopes of the absorbance curves as described above.

#### Determination of total protein content in spirulina extracts

Total protein content was determined in spirulina extract samples exposed to different conditions or processing stages, to evaluate the effect of the intrinsic and extrinsic enzymatic processes to the final nutritional value of the microalgae. Total protein content was determined for fresh spirulina extracts, kept at 4 °C for 1 week (to evaluate possible effect of sample storage) and spirulina extracts submitted to enzymatic reaction with HRP. The protein content of spirulina extracts submitted to extrinsic enzymatic reaction were assessed in three different moments of the enzymatic reaction, identified based on the analysis of the extract degradation profiles, i.e. before enzymatic treatment, after 1 h (at the end of the first process stage) and 24 h (at the end of the second process stage) of enzymatic reaction, following the procedures described below.

Protein solutions of bovine serum albumin (BSA) with different concentrations up to 200 μg mL^−1^ were prepared. An amount of 0.1 mL of each solution was added to individual test tubes. The volume in the test tubes was adjusted to 1 mL with phosphate buffer (100 mmol L^−1^), at pH 7.5. The total protein was determined using a total protein kit through a test tube protocol (TP0100, Sigma‐Aldrich). The Coomassie dye reagent was diluted with water at a 1:4 volume ratio. Then, 2.5 mL of the dye reagent was added to each tube containing 50 μL of extract sample and the mixture was vortexed to ensure homogenisation. The absorbance was measured at a wavelength of 595 nm, using the microplate reader mentioned above, after 2 min of incubation, at room temperature. The protein concentrations were plotted against the corresponding absorbance, resulting in a standard curve, which was then used to determine the total protein content in the spirulina extracts.

#### Statistical analysis

Each experimental condition was studied in triplicate (*n* = 3). Each replicate consisted of an extract from an independent cultured and processed batch of *Limnospira platensis* biomass. The results are expressed as the mean value ± standard deviation of the independent replicates. To determine whether the observed differences between experimental groups were statistically significant, comparative analyses were conducted. The errors were determined using the descriptive statistic in the Data Analysis toolbox of Excel considering a confidence level of 95%.

## RESULTS

### Characterisation of spirulina extracts

**Figure 1:**
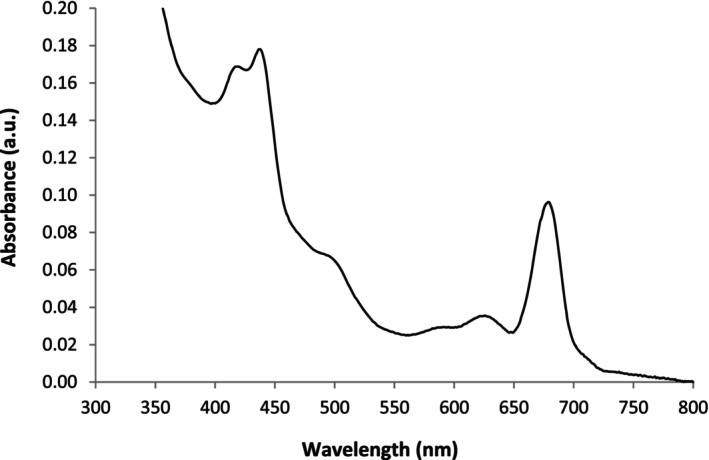
Representative absorbance spectra obtained for the spirulina extracts.

Microalgae have a complex metabolic system, including natural pigments^20^ and diverse proteins, such as chlorophylls, carotenoids and phycocyanin,^6^ which have a vital role in algal photosynthesis and photoprotection. These components are commonly characterised by the presence of absorbance bands located in distinct regions of the visible spectrum and thus contributing to the intense colour generally found in solutions of algae and their derivatives, such as the spirulina extracts. All the spirulina extracts used in the present work were characterised in terms of their total protein content, pH and conductivity. The presence of pigments was elicited based on the analysis of the visible absorbance spectra of the spirulina extracts from different batches. A representative absorbance spectrum obtained for the spirulina extracts used in the present work is illustrated in Fig. 1. The spirulina extracts showed reproducible absorbance spectra, characterised by the presence of four main bands (Fig. 1). The first band located in the 400–450 nm range exhibits a shoulder at 425 nm and a maximum at 440 nm. The second and third bands were less prominent showing maximum absorbances at 500 and 625 nm, respectively. Finally, an intense band was found in the 650–750 nm region showing maximum absorbance at 677 nm. The absorbance spectra obtained for spirulina are in close agreement with spectra of pure chlorophyll, carotenoids and phycocyanin solutions described in previous literature.^3^, ^9^ According to Munawaroh *et al*., the absorbance spectrum of chlorophyll *a* is characterised by peaks at 430 and 660 nm, whereas chlorophyll *b* shows maximum absorbance at 470 and 620 nm.^3^ The small deviations found between the locations of the maximum peak obtained in this work and those published in previous literature may be attributable to the high chemical complexity of the microalgal growth media. The presence of multiple absorbing species, e.g. pigments different from chlorophylls or other species with less relevancy, may result in partial overlapping of the spectral bands,^9^ causing an apparent shift in chlorophyll's maximum absorbance. Despite these small shifts in the maximum absorbance, the spectra obtained in the present work were compared with those published by other authors. This comparative spectral analysis allowed us to ascribe the peaks at 440 and 677 nm to chlorophyll. The band at 440 nm might result from a combined contribution of chlorophylls *a* and *b*, since it is located between the maximum absorbances of these two pigments, expected at 430 and 477 nm, respectively. The peak at 677 nm, although it may potentially include a contribution from other pigments, e.g. chlorophyll *b*, it should predominantly be due to the contribution of chlorophyll *a*. The maximum absorbance found at 625 nm might also be due to chlorophyll *b* and/or to phycocyanin, which are described to present a maximum absorbance at 620 nm.^3^, ^12^ Carotenoids are reported to have a characteristic absorbance band between 400 and 500 nm.^21^ Therefore, they may not only justify the presence of the additional band with maximum absorbance at 500 nm detected in the spectra, but also a possible overlapping with the band from chlorophylls at 440 nm. Despite the possible spectral superimposition from the different pigments present in spirulina extracts, the greenish colour of the microalgal extracts suggested the predominance of chlorophyll pigments. For this reason, and for the sake of simplicity, the changes observed in this spectral region from 400 to 700 nm were mainly attributed to chlorophyll pigments, but keeping in mind the possible contribution from other pigments, as discussed above. In this regard, the spectra obtained for the spirulina extracts not only confirm the presence of hydrophobic pigments, such as chlorophylls *a* and *b*, but also suggest the presence of hydrophilic pigments, such as phycocyanin.

### Effect of physicochemical media conditions on degradation of spirulina extracts

**Figure 2:**
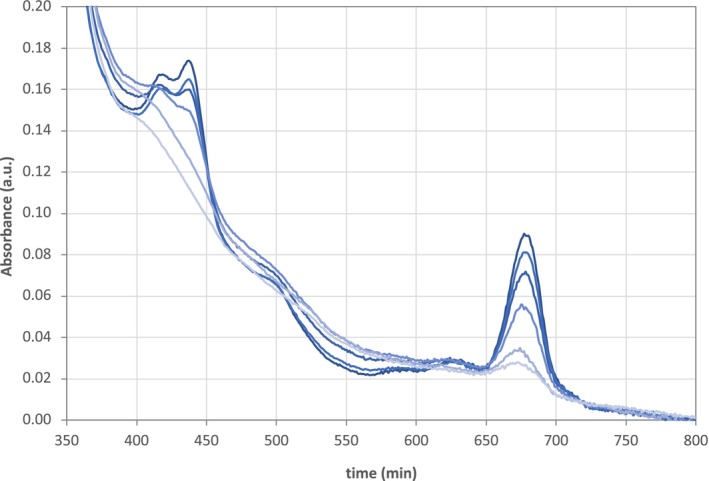
Evolution of the absorbance of spirulina extracts at pH 9, in the visible region of the spectra. The absorbance spectra shown in the plot were collected at 0, 50, 360, 720, 1380 and 1440 min, presented in this order from dark blue to light blue lines.

The stability of the spirulina extracts (at pH 9) exposed to atmospheric conditions was evaluated based on the changes observed in the visible region of the spectra acquired for the spirulina extracts over time. As illustrated in Fig. 2, the absorbance decreased progressively in the whole visible region of the spectra. However, the absorbance decrease was more prominent in the absorbance bands with maximum values centred at 440 and 677 nm, corresponding to the chlorophyll signal. These bands showed low stability, fading during the experiment time. The disappearance of the absorbance bands was attributed to the oxidation of the chlorophyll pigments upon contact with atmospheric oxygen species, light exposure and due to the intrinsic enzymatic mechanisms of spirulina, leading to the formation of colourless products.^17^

#### Effect of spirulina extract media pH on pigment stability

**Figure 3:**
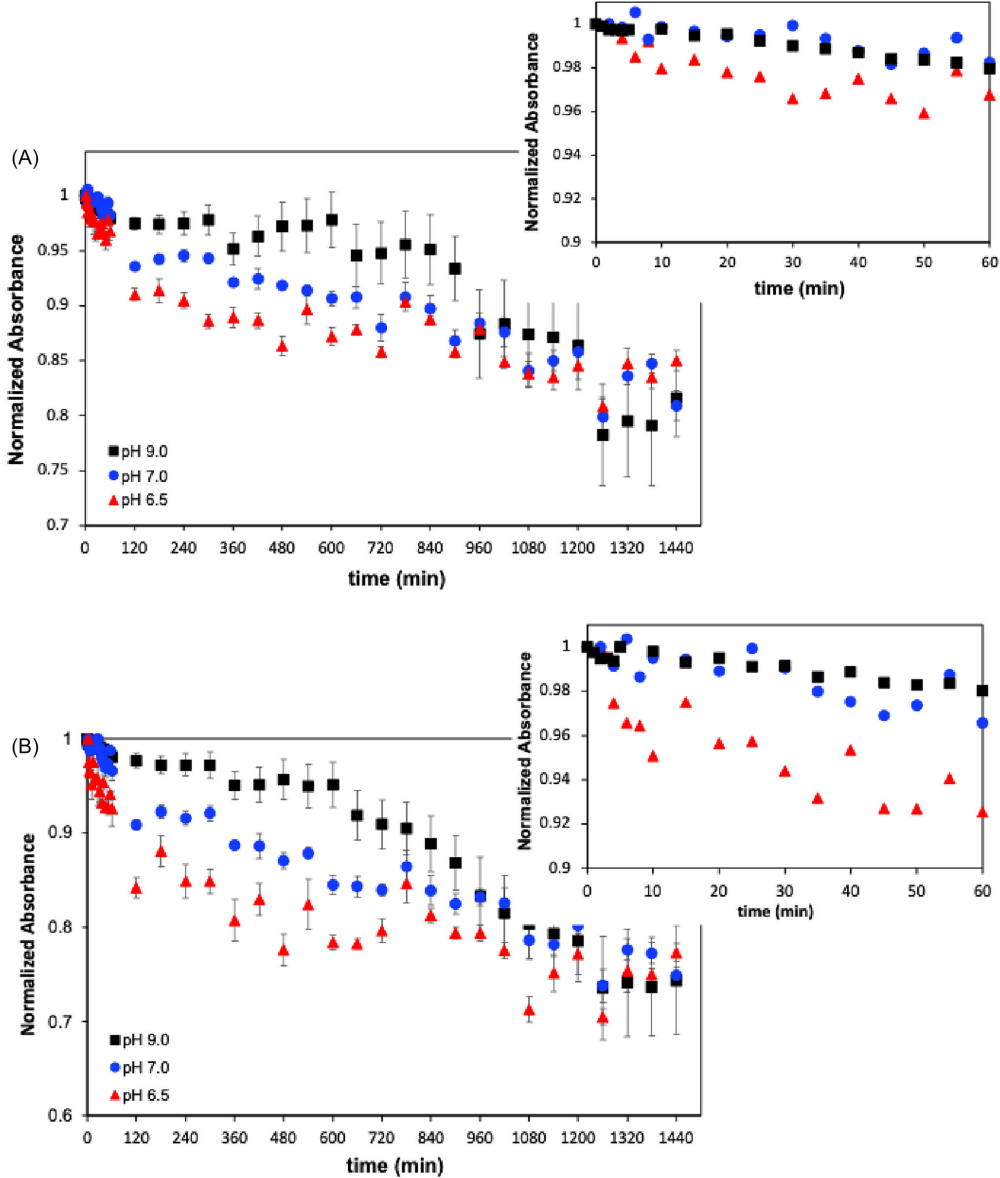
Evolution of spirulina extract absorbance at (A) 440 nm and (B) 677 nm over time, at pH 6.5, 7.0 and 9.0. All spectra were normalised considering the initial absorbance value for *t* = 0 min.

The effect of pH on the stability of the spirulina extracts was evaluated for the same period. This comparative analysis took mainly into account the absorbance changes monitored at 440 and 677 nm. As discussed before, changes in these absorbance bands not only reflect the degradation of chlorophyll pigments, but also correspond to the regions showing higher sensitivity to the extract degradation. Figure 3 shows the evolution of the absorbance values at the above‐mentioned wavelengths over 1440 min obtained for the spirulina extracts at pH 6.5, 7 and 9 (natural pH of the spirulina extracts). The analysis of the spectral changes revealed that the absorbance decrease follows a two‐stage regime. The extract degradation is characterised by a more intense absorbance decline over the first period of 60 min (first stage) more perceptible at the more acidic pH (pH 6.5), followed by a slower absorbance decrease in the second stage. Identical absorbance profiles were observed at 440 nm (Fig. 3(A)) and 677 nm (Fig. 3(B)). However, a slightly higher absorbance decline was observed at 677 nm, which may suggest a higher sensitivity of the pigments absorbing in this spectral region, e.g. chlorophyll *a*, to the oxidising conditions.

In the first stage, the loss of absorbance was found to be higher at pH 6.5 and lower in alkaline conditions, reaching values of 3–6% at pH 6.5 and less than 3% at pH 7 and pH 9. The effect of pH on the absorbance of spirulina extracts was also evident by analysis of the absorbance decreasing rates observed in the first process stage (*t* < 60 min) at the different pH values (Table 3). Higher absorbance decreasing rates were observed at the lower pH studied (pH 6.5). The effect of pH on the stability of the spirulina extracts became more evident at an intermediary stage, with the absorbance decrease reaching 20%, 15% and 10% at pH values of 6.5, 7.0 and 9.0, respectively, after 800 min of experiment time. These differences completely fade away after this period resulting in an identical overall absorbance loss of 25% at the three different pH values, after 1440 min.

**Table 3:** Absorbance decreasing rates for spirulina extracts at different pH values, at 440 nm and 677 nm, in the first stage (*t* < 60 min)

pH	Absorbance decreasing rates (×10^−4^ arb. units. min^−1^)	
	440 nm	677 nm
6.5	−6.71 ± 0.59	−7.28 ± 1.10
7.0	−2.93 ± 0.25	−6.51 ± 0.58
9.0	−3.30 ± 0.12	−3.26 ± 0.22

As mentioned above, the observed absorbance decrease may be due to oxidation mechanisms resulting from the contact of the pigments, such as chlorophyll, with oxidising species (e.g. oxygen species) formed in the microalgal extracts and those present in the air. Also, it may be ascribed to the activity of specific enzymes intrinsic to the composition of the spirulina extracts. In agreement with the obtained results, the degradation of chlorophyll has been described in previous publications as a two‐stage process resulting from the metabolic activity of enzymes naturally present in chlorophyll‐containing species, such as hydrolase (chlorophyll‐chlorophyllido Chlase, chlorophyllase), peroxidases (e.g. chlorophyll degrading peroxidase), oxygenases (e.g. pheide a oxygenase) and reductases (e.g. red chlorophyll catabolite reductase), all involved in the natural chlorophyll degradation pathway during plant senescence or oxidative stress.^17^, ^18^

For instance, the enzymatic activity of chlorophyll‐chlorophyllido Chlase involves the formation of greenish products and occurs with the cleavage of the tetrapyrrole macrocyclic ring. The reactions taking place in the later stage involve the cleavage of the macrocyclic ring of tetrapyrrole followed by the conversion of the early formed products to colourless compounds.^17^ Alkaline conditions are known to be the ideal for the development and maintenance of spirulina^6^, ^7^ as they promote the synthesis of a large array of antioxidant compounds, such as chlorophyll *a*, carotenoids and phycocyanin.^22^ The increase of the microalgal antioxidant content not only enriches the nutritional value of the extract, but also confers protection against oxidative stress conditions, due to the radical scavenging activity. Hence, the antioxidant activity in spirulina microalgae is pH‐dependent and it was found to be higher at pH 9, which may possibly explain the higher resistance of the spirulina extracts to colour degradation at this pH.^22^ This effect was also confirmed in the present work, since a higher absorbance loss was observed at lower pH values (pH 6.5).

#### Effect of sonication on stability of spirulina extracts

**Figure 4:**
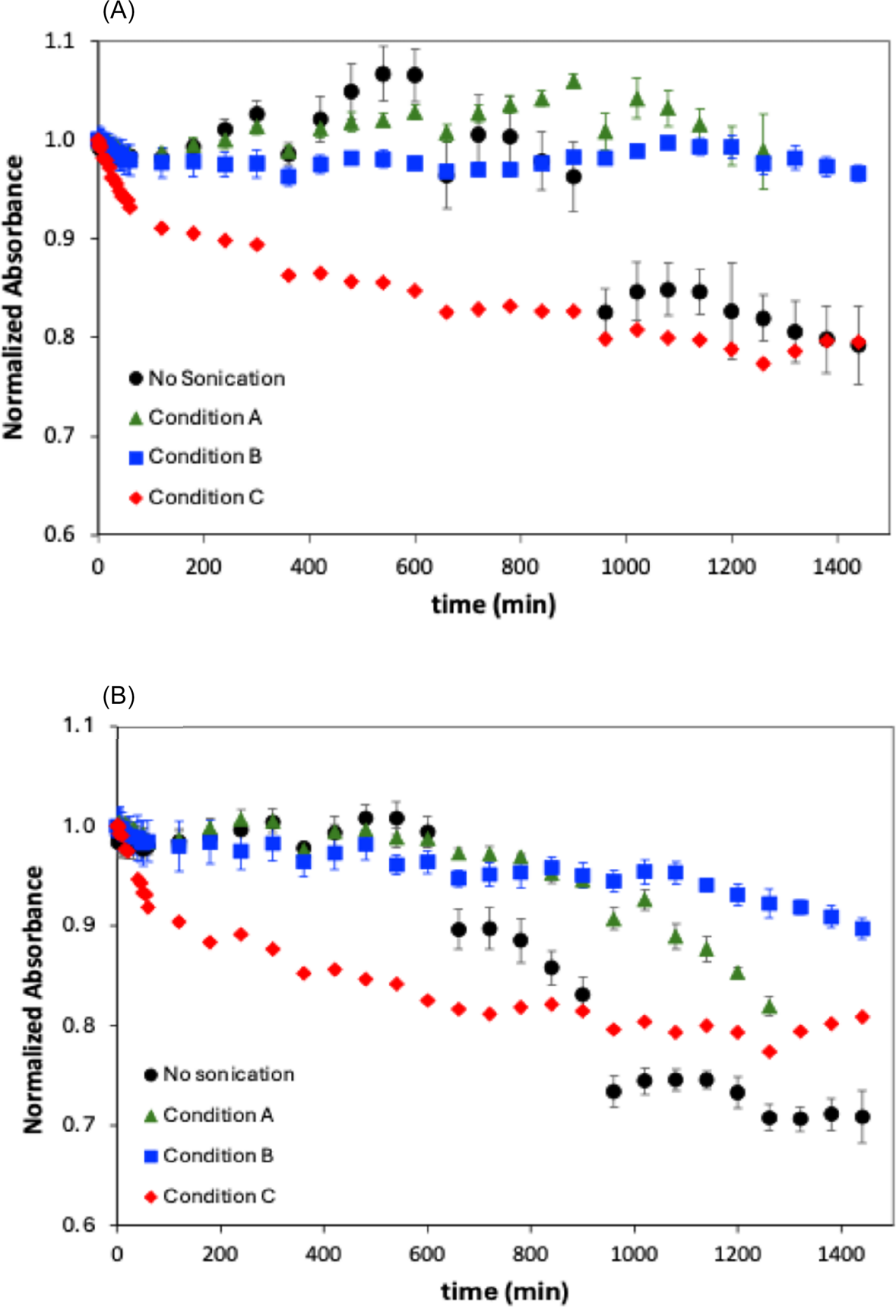
Evolution of spirulina extract absorbance at (A) 440 nm and (B) 677 nm over time, before and after sonication.

The efficiency of pigment degradation depends highly on the amount of oxygen species present in solution and the accessibility of these species and enzymes (generally allocated in the chloroplast subfractions, e.g. chloroplast envelope and inner membranes) to the pigments.^18^ Therefore, it is plausible to consider that pigment degradation may be enhanced by stirring conditions. Higher stirring rates would promote the dissolution of oxygen in the spirulina extracts, while leading to the release of enzymes and pigments enclosed in the vacuoles or contained within the chloroplast fragments (still present in the microalgal extracts), thus increasing their access to the pigments. In this regard, studies were performed to evaluate the ability to improve the degradation of pigments in the spirulina extracts by sonication. Spirulina extracts were exposed to the sonication conditions described in Table 2. Analysis of the absorbance spectra at 440 and 677 nm was carried out to assess the effect of sonication on pigment degradation, i.e. chlorophyll. Three different sonication conditions, established by variation of times and amplitudes, were analysed. The first condition, referred to as sonication A, was the least intense condition, and involved a sonication time of 2 min with an amplitude of 20%. Sonication B consisted of a sonication time of 2 min with an amplitude of 40%, whereas sonication C involved a sonication time of 6 min with an amplitude of 40% and corresponded to the most intense condition. The specific on/off pulse cycles for each condition are detailed in Table 2. The observed spectral changes are depicted in Fig. 4, showing no major differences in the spectral profiles acquired for non‐sonicated spirulina and that exposed to milder sonication conditions A and B (Table 2). The spectral profiles obtained for these spirulina solutions showed complete overlap at least for the initial period of 10–13 h. In contrast, the exposure of spirulina extracts to more intense sonication conditions, i.e. condition C (Table 2), resulted in a faster decrease of the absorbance, particularly evident in the first period of the process (60 min).

A comparative analysis of the absorbance decreasing rates observed in this period for the different sonication conditions, listed in Table 4, shows that more intense sonication conditions (condition C) led to absorbance rates one order of magnitude higher than those obtained in milder sonication conditions, thus expressing a faster pigment degradation, in the initial process stage (Fig. 4). In aggrement with that discussed above, these results suggest that the intensification of the sonication conditions enhanced the transport of atmospheric oxygen species into the extract media. On the one hand, this resulted in the increase of the total concentration of oxidising species in the media, which improved the pigment degradation kinetics. On the other hand, it is plausible to think that sonication may have led to the increase of the activity of intrinsic enzymes by increasing their availability in the media or by increasing the frequency of molecular collisions improving pigment degradation (in this case chlorophylls *a* and *b*). However, and despite the faster chlorophyll degradation at the initial stage, the overall absorbance decline obtained for sonicated spirulina extracts reached values of 20%, which was not higher than those found for non‐sonicated spirulina extracts (Fig. 4). The absence of differences in the overall absorbance decline suggests that in later stages the degradation process may be limited by the lower availability of reactants, other than pigments themselves (e.g. co‐factors, co‐substrates) due to their faster consumption or depletion (e.g. enzymatic denaturation due to sonication) in the initial stages of the degradation process.

**Table 4:** Absorbance decreasing rates for spirulina extracts at 440 and 677 nm, in the first stage (*t* < 60 min), upon exposure to sonication

Sonication	Absorbance decreasing rates (×10^−4^ arb. units. min^−1^)	
	440 nm	677 nm
Non‐sonicated	−0.98 ± 0.13	−1.75 ± 1.52
Condition A	−2.66 ± 0.10	−3.08 ± 0.33
Condition B	−3.83 ± 0.22	−3.13 ± 0.14
Condition C	−11.1 ± 0.35	−13.3 ± 0.37

#### Effect of hydrogen peroxide

**Figure 5:**
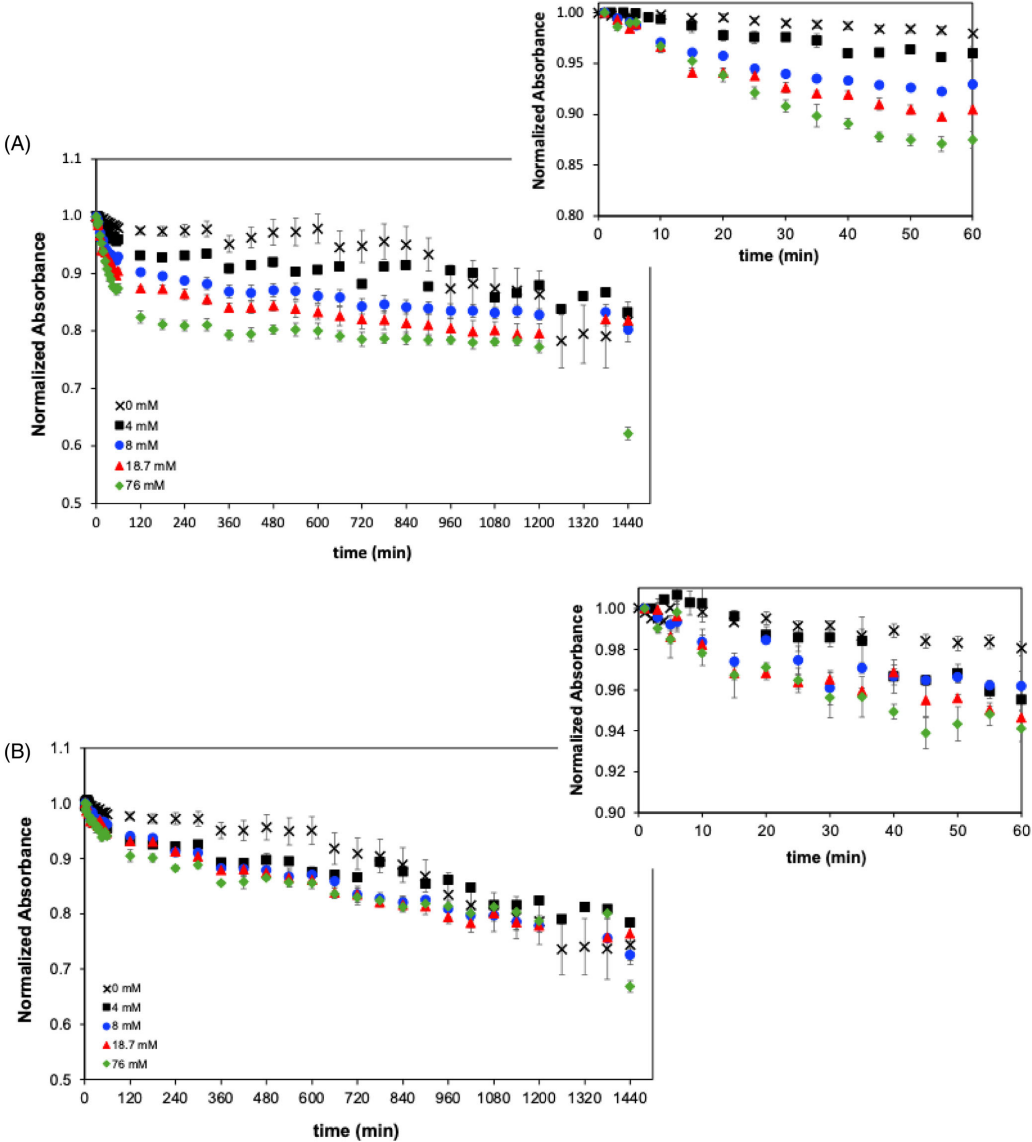
Evolution of spirulina extract absorbance at (A) 440 nm and (B) 677 nm over time, at different hydrogen peroxide concentrations: 0, 4, 8, 18.7 and 76 mmol L^−1^. All spectra were normalised considering the initial absorbance value at *t* = 0 min.

Hydrogen peroxide (H_2_O_2_) is a strong oxidising agent, largely used as an electron acceptor in enzymatic reactions by oxidoreductases, oxygenases, reductases and hydrolases, typically involved in oxidative stress in plants,^18^ and thus acts as an enhancer of the enzymatic reactions catalysed by these enzymes. In this regard, the possible effect of H_2_O_2_ in promoting pigment degradation by enzymes intrinsic to spirulina extracts was assessed in this work. The impact of different H_2_O_2_ concentrations (0, 4, 8, 18.7 and 76 mmol L^−1^) (Fig. 5) on the absorbance spectra of spirulina extracts was studied.

A comparative analysis of the absorbance profiles showed that the presence of H_2_O_2_ led to a higher absorbance decrease, clearly evidencing an improved pigment degradation. This effect is particularly noticiable in the first stage of the degradation process where the increase of H_2_O_2_ concentration from 0 to 76 mmol L^−1^ led to an increase of the absorbance decline rates by one order of magnitude, as listed in Table 5. The H_2_O_2_ effect was more accentuated at 440 nm than at 677 nm, resulting in absorbance decreases of 8.8% to 28.3% over the first stage (*t* < 60 min) at 440 nm. In the second stage, the absorbance decline observed at 677 nm was slightly more pronounced than that observed at 440 nm, resulting in a similar absorbance decrease, of ~30%, after 24 h, at both wavelengths. The differences observed in the absorbance decreasing rates at 440 and 677 nm, over the two process stages, may be attributable to a different susceptibility of the compounds contributing to these two different spectral regions. For instance, one may hypothesise that the faster decrease at 440 nm in the first stage may be due to a higher sensitivity of chlorophyll *b* than chlorophyll *a* (considering that the band at 677 is mainly due to chlorophyll *a*) to oxidation by H_2_O_2_ or to a higher sensitivity of pigments such as carotenoids which is expected to have an important contribution to the absorbance band at 440 nm.

**Table 5:** Absorbance decreasing rates for spirulina extracts at 440 and 677 nm, in the first stage (*t* < 60 min), by adding different concentrations of H_2_O_2_

[H_2_O_2_] (mmol L^−1^)	Absorbance decreasing rates (×10^−4^ arb. units. min^−1^)	
	440 nm	677 nm
0	−3.13 ± 0.13	−3.10 ± 0.24
4.0	−8.89 ± 0.45	−9.30 ± 0.39
8.0	−19.6 ± 1.40	−7.48 ± 0.62
18.7	−18.9 ± 1.07	−8.56 ± 0.81
76.0	−28.3 ± 1.10	−12.2 ± 1.10

### Degradation of spirulina extracts by extrinsic enzymatic reaction

**Figure 6:**
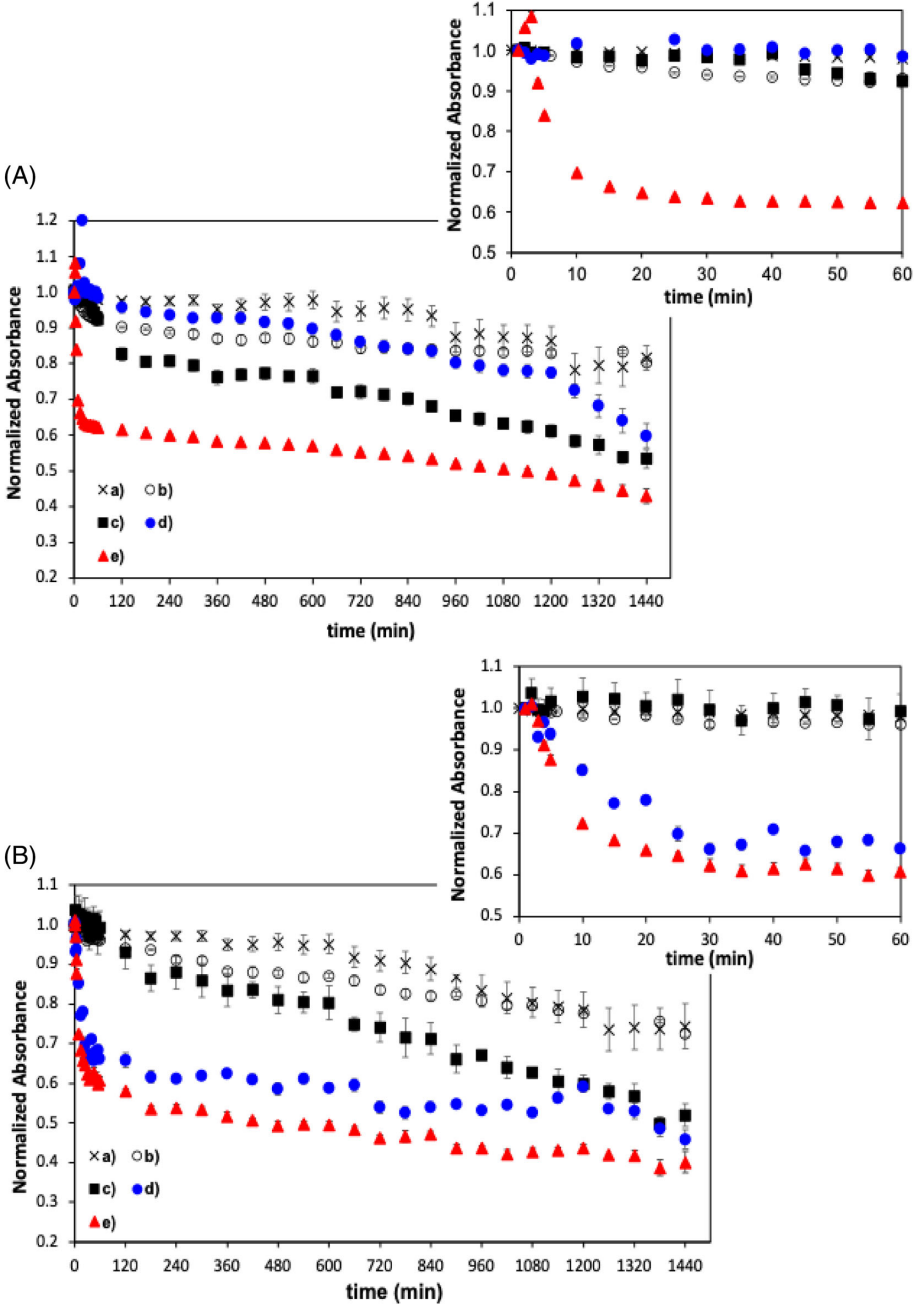
Evolution of spirulina extract absorbance at (A) 440 nm and (B) 667 nm over time: (a) spirulina extract, (b) spirulina extract with addition of 8 mmol L^−1^ of H_2_O_2_, (c) spirulina extract with addition 7.1 μmol L^−1^ of HRP and 8 mmol L^−1^ of H_2_O_2_, (d) spirulina extract with addition of 7.1 μmol L^−1^ of HRP, 8 mmol L^−1^ of H_2_O_2_ and 3 mmol L^−1^ of PSA and (e) spirulina extract with addition of 7.1 μmol L^−1^ of HRP, 8 mmol L^−1^ of H_2_O_2_ and 5.8 mmol L^−1^ of PSA. Enlarged views up to 60 min are shown in the insets. All spectra were normalised considering the initial absorbance value at *t* = 0 min.

Chlorophyll is one of the main components in spirulina and it is not readily bleached *in vitro* by the action of intrinsic enzymes, in the presence or absence of hydrogen peroxide.^23^ The possibility to accelerate the degradation of spirulina extracts by the addition of an enzyme extrinsic to the spirulina extract composition was also evaluated. HRP was selected for this purpose and used as described in the Experimental section. As shown in Fig. 6, the enzymatic activity of HRP in spirulina extracts results in a two‐stage process identical to that found for spirulina degradation by intrinsic oxidation mechanisms, i.e. a more accentuated absorbance decline over the first process stage (*t* < 60 min), followed by a smoother decline of the absorbance in a later stage. However, the addition of HRP led to a more intense absorbance decline in the first process stage than that obtained by intrinsic degradation. The pigment degradation due to extrinsic enzymatic activity (in the presence of H_2_O_2_ and 5.3 mmol L^−1^ of PSA) led to an absorbance decrease of 40% whereas an absorbance decline of <15% was obtained due metabolic activity intrinsic to spirulina extracts (i.e. in the absence of HRP), either upon sonication (Fig. 4) or in the presence of H_2_O_2_ (Fig. 5).

The effects of H_2_O_2_ and PSA concentrations on the degradation of spirulina extracts were also studied. It was found that the decrease of the absorbance was higher with the increase of H_2_O_2_ and PSA concentrations, thus not suggesting the existence of a potential inhibiting effect by the H_2_O_2_ on the HRP activity, in the conditions used in the present work.^24^

Table 6 presents the absorbance decreasing rates observed in the initial stage (*t* < 60 min) of pigment degradation, in spirulina extracts, after addition of HRP with 8 mmol L^−1^ of H_2_O_2_ and different concentrations of PSA. A comparative analysis of the results listed in Table 6 showed that the presence of PSA led to an increase of the degradation rates by two orders of magnitude in the initial reaction period, leading to a 40% loss of the absorbance intensity in the presence of 5.8 mmol L^−1^ of PSA, whereas a decrease of less than 10% was registered in the absence of PSA, in the same period (Fig. 6).

**Table 6:** Absorbance decreasing rates for spirulina extracts at 440 and 677 nm, in the first stage (*t* < 60 min), in the presence of extrinsic peroxidase (HRP), with and without the PSA co‐factor

Enzymatic reaction conditions	Absorbance decreasing rate (×10^−4^ arb. units. min^−1^)	
	440 nm	677 nm
7.1 μmol L^−1^ HRP + 8 mmol L^−1^ H_2_O_2_	−12.0 ± 0.68	−8.62 ± 0.69
7.1 μmol L^−1^ HRP + 8 mmol L^−1^ H_2_O_2_ + 3 mmol L^−1^ PSA	−1.99 ± 0.06	−117.81 ± 8.85
7.1 μmol L^−1^ HRP + 8 mmol L^−1^ H_2_O_2_ + 5.8 mmol L^−1^ PSA	−477.78 ± 106.6	−351.53 ± 24.49

The differences found in the first process stage were mostly dissipated after 1440 min (24 h), due to different degradation rates registered in the second stage for each condition, i.e. HRP with H_2_O_2_ only, HRP with H_2_O_2_ at 3 mmol L^−1^ and 5.8 mmol L^−1^ of PSA (Fig. 6). In fact, the decrease of the absorbance was more pronounced during the second stage of the process, for samples without PSA, in contrast to that observed in the presence of this co‐substrate. The lower decrease of absorbance in the second stage, in the presence of PSA, may be attributed to a significant reduction of the pigment concentration (the enzymatic substrate), to a loss of enzymatic activity or to the consumption of PSA over the first process stage. The more accentuated decrease of the absorbance in spirulina extracts without PSA, during the second stage, partially compensated the slower decrease found in the initial period, in these samples, rendering a global absorbance decrease of 48%, in 24 h. Still it was smaller than the absorbance decrease of 60 % observed in the presence of PSA. For comparative reasons, 18 h would be needed for spirulina extracts without PSA to reach the absorbance loss obtained in 1 h for the same extracts in the presence of 5.8 mmol L^−1^ PSA.

Similar interpretation resulted from the analysis of the spectra obtained at 440 nm, suggesting that chlorophylls *a* and *b* exhibit identical degradation behaviour when submitted to enzymatic degradation by extrinsic peroxidase (HRP).

Considering the potential application of spirulina extracts as food additive or nutraceutical ingredient it becomes also important to evaluate the effect of these enzymatic processes on the nutritional value of the spirulina extracts. A preliminary analysis of the effect of the enzymatic process on the nutritional value was conducted based on the variation of the total protein content due to the enzymatic process.

The total protein concentration was determined using the Bradford assay^25^ for spirulina extracts after 1 week of storage at 4 °C and for those submitted to enzymatic degradation with HRP, after the first reaction stage (*t* < 60 min) and at the end of 24 h. The protein loss was assessed taking the protein content in fresh samples as the reference value.

As presented in Table 7, only a small decrease in the total protein content, less than 5%, was observed in all cases. This minor loss is likely associated with protein denaturation or protein adsorption to insoluble components present in the spirulina extract, and subsequent protein precipitation. These results suggest that the pigment degradation by an extrinsic peroxidase does not significantly affect the nutritional value of the spirulina extracts. However, additional analysis of a potential effect of HRP treatment on the content of other components, e.g. carotenoids, which also contribute to the nutritional value of spirulina, is required to further support this conclusion.

**Table 7:** Percentage change of protein content in spirulina extracts during 1 week of storage at −4 °C and upon enzymatic degradation with HRP after the first process stage (*t* = 1 h) and the end of the second process stage (*t* = 24 h)

Sample	Time	Protein loss (%)
Spirulina extract A	1 week storage	3.83 ± 0.78
Spirulina extract B		7.46 ± 0.43
Spirulina extract C		2.82 ± 0.12
Extrinsic enzymatic degradation	First stage (1 h)	0.66 ± 0.38
	Second stage (24 h)	3.97 ± 0.66

## CONCLUSIONS

Microalgal extracts, such as *Limnospira platensis* extracts, are important sources of food ingredients and nutraceutical compounds finding relevant application in the food and pharmaceutical industries. Constraints to the use of these extracts as food ingredients have been attributed to a low consumer acceptance explained by the intense colour of these extracts. The development of efficient pigment degradation strategies is thus required to improve the commercialisation of microalgal extracts.

This work aims to evaluate the efficiency of exogenous biocatalytic processes using HRP for discolouration of spirulina extracts compared with pigment degradation due to oxidation mechanisms associated with the intrinsic metabolism of these microalgae. Pigment degradation was elicited based on the decrease of the absorbance in the visible region of the spectra and thus associated with extract decolouration. Intrinsic and extrinsic degradation of the microalgal extracts led to the decrease of the absorbance intensity in the whole visible range of the spectra, but more perceptible for the absorbance bands with maximum intensity at 440 and 677 nm. These spectral bands were mainly ascribed to chlorophylls *a* and *b*, and thus correlated with the degradation of these pigments. Pigment degradation in spirulina extracts, due either to intrinsic metabolic activity of spirulina or to extrinsic enzymatic reaction by HRP, was characterised by a two‐stage process, showing a faster absorbance decrease in the initial period of 60 min (first stage) followed by a slower absorbance decrease during the second stage of the process. The intrinsic metabolism of microalgae associated with the presence of oxidising agents (e.g. oxygen species) or with the enzymes naturally present in spirulina extracts led to very modest absorbance decrease during the first process stage, less than 6%, and an overall absorbance decrease not greater than 25% was observed after 24 h. Slightly improved absorbance decreasing rates were obtained during the first stage by adjustment of extract pH to more acidic conditions, by extract sonication and by the presence of an electron acceptor (H_2_O_2_), leading to an absorbance decrease varying between 10% and 20% in the first process stage. Nevertheless, these changes were attenuated during the second stage resulting in a similar overall absorbance decrease not higher than 25% at the end of 24 h.

The addition of HRP led to a more significant decrease of the absorbance, more evident in the presence of PSA, which was used as the enzymatic reaction co‐substrate. In this case, the absorbance decreasing rates increased significantly in the first stage resulting in absorbance losses of ~40% in less than 30 min, contrasting with poor decreasing values of <6% obtained for the same period in the absence of HRP, at pH 9.

Enzymatic processes based on the use of peroxidases, such as HRP, allowed an efficient degradation of microalgal extracts at the laboratory scale without significantly affecting the protein content. This proof‐of‐concept study establishes the fundamental efficacy of the approach. However, its translation into the food or pharma industries primarily requires a rigorous assessment of toxicological and safety profiles of all the substances used in the process, as well as a complete study on the effect of the enzymatic decolouration process on the nutritional value of the extracts. Hydrogen peroxide poses relative concern as it decomposes spontaneously into water and molecular oxygen, leaving no residual traces.^26^ However, since its decomposition is not instantaneous, its persistency in the extract should be carefully analysed for consequent optimisation of the enzymatic decolouration conditions. HRP is intended for use as a processing aid and not as a food additive. Adequate downstream separation technologies, such as membrane filtration or strategies involving the development of integrated enzymatic separation processes should be considered in future studies, allowing for the removal of the enzyme from the final extract, and its subsequent re‐use. The choice of the phenolic co‐substrate is the most critical aspect. In this proof‐of‐concept study, PSA was employed as a co‐substrate for robust activation of the enzymatic mechanism. For any commercial application, this component must be replaced by a natural, food‐grade phenolic compound.

Industrial applicability is based on the ability to solve critical challenges related to economic feasibility and scalability. The potential for industrial application of this enzymatic decolouration process hinges on the long‐term stability and reusability of the peroxidase enzyme. While the present study proved a good enzymatic efficacy in single‐enzyme use, future work will focus on enzyme stabilisation strategies, i.e. enzyme immobilisation in solid supports, e.g. micro‐ to nanocarriers and membranes to enable catalyst recovery and reuse across multiple cycles, thereby improving cost‐effectiveness, paving the way for improved commercialisation viability.

## FUNDING INFORMATION

PTDC/CTM‐CTM/31924/2017 – POCI‐01‐0145‐FEDER‐031924 – FCT, Fundação para a Ciência e a Tecnologia, IP. UID/50006/2025 – FCT, Fundação para a Ciência e a Tecnologia, IP. UID/PRR/50006/2025 – FCT, Fundação para a Ciência e a Tecnologia, IP. LA/P/0008/2020 – FCT, Fundação para a Ciência e a Tecnologia, IP.

## Data Availability

The data that support the findings of this study are available from the corresponding author upon reasonable request.
